# Long‐term survival with systemic therapy in the last decade: Can melanoma be cured?

**DOI:** 10.1111/1346-8138.17147

**Published:** 2024-02-15

**Authors:** Kenjiro Namikawa, Eiji Nakano, Dai Ogata, Naoya Yamazaki

**Affiliations:** ^1^ Department of Dermatologic Oncology National Cancer Center Hospital Tokyo Japan

**Keywords:** BRAF, immunotherapy, melanoma, survival, targeted therapy

## Abstract

Immune checkpoint inhibitors have been shown to prolong survival of patients with several types of cancer, and the finding was first established in melanoma. Previously, systemic therapy for advanced melanoma aimed only at tumor control and palliation of symptoms. However, in recent years, some patients who received systemic therapy have achieved a complete response and survived without continuous treatment for more than several years. This review discusses the long‐term survival rates achieved with currently used systemic therapies and their future perspectives. Long‐term survival is currently most likely to be achieved with the use of the standard‐dose combination of nivolumab plus ipilimumab, however, this regimen is associated with a high frequency of serious or persistent immune‐related adverse events. Several new anti‐PD‐1‐based combination therapies with a better risk–benefit balance are currently under development. Although the acral and mucosal subtypes tend to be less responsive to immune checkpoint inhibitors, anti‐PD‐1‐based combination therapy should continue to be investigated for these subtypes owing to its potential for better long‐term survival. With the development of efficacious immunotherapy and targeted therapy, it is important to determine the optimal duration of systemic therapy to avoid unnecessary health and financial burdens as well as to improve efforts to support long‐term cancer survivors. As the goal of systemic therapy shifts from tumor control to long‐term survival, in future clinical trials, long‐term clinical outcomes should be evaluated to assess the benefits of novel agents.

## 1 INTRODUCTION

Immune checkpoint inhibitors (ICIs) have been shown to prolong survival of the patients with several types of cancer, and the finding was first established in melanoma.^1^ Nivolumab, an anti‐PD‐1 antibody (anti‐PD‐1), was first approved in Japan in July 2014, followed by pembrolizumab, in 2016, and nivolumab in combination with ipilimumab, an anti‐CTLA‐4 antibody (anti‐CTLA‐4), was approved in 2018. BRAF/MEK inhibitors (BRAF/MEKi) have also been shown to prolong the survival of patients with advanced BRAF V600‐mutant melanoma compared with BRAF inhibitor monotherapy. BRAF/MEKi, such as dabrafenib plus trametinib (Dab/Tram) and encorafenib plus binimetinib (Enco/Bini), were approved in Japan in 2016 and 2019, respectively. Both ICIs and BRAF/MEKi have become the standard of care for melanoma. Previously, systemic therapy for advanced melanoma was aimed only at tumor control and palliation of symptoms. However, in recent years, some patients who received systemic therapy have achieved a complete response (CR) and have survived without continuous treatment for several years. Ten years have passed since anti‐PD‐1 was first approved. This review discusses the long‐term survival rates achieved with currently used systemic therapies and their future perspectives.

## 2 LONG‐TERM OUTCOMES OF IMMUNOTHERAPY AND TARGETED THERAPY

### 2.1 Single‐agent immunotherapy

Previous trials of first‐line nivolumab include the CheckMate 066 and the ONO‐4538‐08 trials. In CheckMate 066, a randomized phase III trial, nivolumab was compared with dacarbazine (DTIC) in treatment‐naïve patients with BRAF wild‐type advanced melanoma.^2^, ^3^, ^4^, ^5^ The ONO‐4538‐08 trial was a single‐arm, Japanese phase II study of nivolumab for treatment‐naive patients with advanced melanoma.^6^, ^7^ The 5‐year progression‐free survival (PFS) and overall survival (OS) rates of nivolumab‐treated patients from the CheckMate 066 trial were 28% and 39%, respectively, indicating a sustained long‐term response^5^ (Table 1). Considering that the 5‐year PFS and OS rates of the DTIC‐treated patients were only 3% and 17% respectively, nivolumab has been shown to have better long‐term efficacy. The ONO‐4538‐08 trial in Japan reported 5‐year PFS and OS rates of 17% and 26%, respectively,^7^ which are 10% lower than those in the CheckMate 066 trial. The reason for the inferior response to ICIs in Asian patients compared to Caucasian patients may be the higher frequency of acral and mucosal subtypes, which have a lower tumor mutational burden (TMB).^8^, ^9^ Of the 24 participants in the ONO‐4538‐08 trial, seven (29%) had the acral subtype and six (25%) had the mucosal subtype. In addition, clinical responses to ICI treatment of non‐acral cutaneous melanoma are lower in Asian patients, possibly due to their skin type being less sensitive to ultraviolet (UV) light, which may result in lower UV‐induced TMB.^10^ However, there are no data to confirm racial differences in the TMB of non‐acral cutaneous melanoma. A 5‐year follow‐up analysis for the ONO‐4538‐08 trial showed that the 5‐year OS of patients with the superficial spreading type of melanoma was 67%, whereas, for patients with the acral and mucosal subtypes, it was 14% and 17%, respectively. However, as some patients with acral and mucosal melanoma achieve long‐term survival, anti‐PD‐1‐based immunotherapy is considered the mainstay of systemic therapy, even for these rare subtypes.

**TABLE 1:** Long‐term outcomes of the pivotal phase III and Japanese phase II trials.

Class	Agent	*N*	Treatment duration	CR rate	mPFS	5y‐PFS	Months	5y‐OS	Beyond 5 years	Ref
Clinical trial										
*Anti‐PD‐1*										
CheckMate 066	Nivo	210	Until PD	20%	5.1 mo	28%	37.3 mo	39%	NA	2, 3, 4, 5
KEYNOTE‐006	Pembro	556	Up to 2y	13%	9.4 mo	25%	32.7 mo	40%	7y‐OS: 38%	11, 12, 13, 14
ONO‐4538‐08^a^	Nivo	23	Until PD	9%	5.9 mo	17%	32.9 mo	26%	NA	6, 7
*Anti‐PD‐1/CTLA‐4*										
CheckMate 067	Nivo/Ipi	314	Until PD	22%	11.5 mo	37%	72.1 mo	52%	6.5y‐OS: 49%	15, 16, 17, 18, 19
ONO‐4538‐17^a^	Nivo/Ipi	30	Until PD	7%	NR	NA	NR	NA	NA	20, 21
*BRAF/MEK inhibitors*										
COMBI‐v, −d	Dab/Tram	563	Until PD	19%	11.1 mo	19%	25.9 mo	34%	NA	42, 43, 44, 45, 46
COLUMBUS^b^	Enco/Bini^c^	192	Until PD	14%	14.9 mo	23%	33.6 mo	35%	7y‐OS: 27%	49, 50, 51, 52, 53, 54

Abbreviations: CR, complete response; Dab/Tram, dabrafenib/trametinib; Enco/Bini, encorafenib/binimetinib; mo, months; OS, overall survival; mPFS, median progression‐free survival; N, number of patients; NA, not available; Nivo, nivolumab; Nivo/Ipi, nivolumab/ipilimumab; NR, not reached; PD, progressive disease; Pembro, pembrolizumab; Ref, reference.

^a^ Japanese clinical trial.

^b^ Participants from Japan.

^c^ COMBO450.

Pembrolizumab was compared with ipilimumab in patients with advanced melanoma who had received no more than one previous systemic therapy.^11^, ^12^, ^13^, ^14^ The 5‐year PFS and OS of the KEYNOTE‐006 trial in the pembrolizumab group were 25% and 40% respectively, and the 7‐year PFS and OS were 24% and 38%, respectively. The little difference between the 5‐year and 7‐year outcomes indicates a sustained response of pembrolizumab over time^14^ (Table 1). Notably, patients in the KEYNOTE‐006 trial received pembrolizumab for up to 2 years, whereas in other trials, treatment was continued until progressive disease or unacceptable toxicity was observed. The rarity of progression beyond 2 years in the pembrolizumab treatment group in the KEYNOTE‐006 trial suggested that a treatment duration of 2 years may be sufficient for patients without disease progression.

### 2.2 Combination immunotherapy

For nivolumab plus ipilimumab (Nivo/Ipi) therapy, a standard regimen of nivolumab (1 mg/kg) in combination with ipilimumab (3 mg/kg) every 3 weeks for four doses, followed by maintenance nivolumab was evaluated for treatment‐naïve patients with advanced melanoma in the CheckMate 067 randomized phase III trial,^15^, ^16^, ^17^, ^18^, ^19^ and in ONO‐4538‐17, a Japanese single‐arm phase II trial.^20^, ^21^ The 6.5‐year outcomes of the CheckMate 067 trial have been reported^19^ (Table 1). The incidence of grade 3 or higher treatment‐related adverse events (AEs) with Nivo/Ipi, nivolumab alone, and ipilimumab alone were 59%, 24%, and 28% respectively, and median OS was 72.1, 36.9, and 19.9 months, respectively. Nivo/ipi therapy was associated with the highest frequency of immune‐related AEs (irAEs) and long‐term survival rates.

Because a standard regimen of Nivo/Ipi therapy is associated with a high frequency of irAE, a flipped dose of the Nivo/Ipi regimen (nivolumab 3 mg/kg plus ipilimumab 1 mg/kg) was evaluated in the CheckMate 511 trial,^22^ and reduced cycles (2 cycles) of Nivo/Ipi regimen was evaluated in the ADAPT‐IT trial.^23^ The flipped dose treatment arm in the CheckMate 511 trial showed a similar survival outcome with favorable toxicity compared to the standard dose treatment arm. However, its primary endpoint was safety, and efficacy was only exploratory. The ADAPT‐IT trial showed that most responses occur within the first two cycles. However, because it was a single‐arm phase II study, further studies are needed to confirm if this approach can replace the standard regimen.

Due to the higher frequency of serious irAEs induced by Nivo/Ipi, the situations in which Nivo/Ipi should be selected as first‐line therapy need to be identified. In practice, Nivo/Ipi is likely to be selected for patients who can tolerate irAEs and have the following conditions: asymptomatic brain metastases based on the CheckMate 204^24^, ^25^, ^26^ and ABC trials,^27^ BRAF V600‐mutated melanoma based on the DREAMseq trial,^28^ treatment‐failure after anti‐PD‐1 monotherapy in the adjuvant setting based on a retrospective study^29^ and in the metastatic setting based on a randomized phase II study,^30^ liver metastasis based on retrospective studies,^31^, ^32^ and high lactate dehydrogenase (LDH)/low PD‐L1 expression/mucosal (and acral) subtypes based on subgroup analyses of the CheckMate 067 trial.^18^, ^33^ Of these factors, multicenter retrospective studies in Japan attempted to address whether patients with BRAF V600‐mutant melanoma, acral/mucosal subtypes of melanoma, and non‐acral/mucosal BRAF wild‐type melanoma should receive Nivo/Ipi upfront. For BRAF V600‐mutant melanoma, the clinical outcomes of first‐line BRAF/MEKi, anti‐PD‐1, and Nivo/Ipi therapies were analyzed in 336 Asian patients. Although the superiority of first‐line Nivo/Ipi over BRAF/MEKi appears modest, anti‐PD‐1 was inferior to Nivo/Ipi in terms of both PFS and OS,^34^ consistent with the findings of the subgroup analysis of BRAF status in the CheckMate 067 trial. In contrast, a multicenter, real‐world study of advanced acral,^35^ mucosal,^36^ and non‐acral/mucosal BRAF wild‐type melanomas^37^ found that the clinical outcomes of patients who received first‐line anti‐PD‐1 therapy or Nivo/Ipi therapy were similar. For advanced mucosal melanoma, an international, multicenter, retrospective study also demonstrated similar efficacies of first‐line anti‐PD‐1 and Nivo/Ipi therapies regardless of ethnicity.^38^ However, further investigations to identify biomarkers that can indicate patients who would benefit most from upfront Nivo/Ipi therapy are warranted.

### 2.3 Targeted therapy

The most common genetic alteration in melanoma is the BRAF V600 mutation, which is estimated to account for approximately 50% of all melanomas in Caucasian patients.^39^ In Asian patients, it is 20%–30% as they are more likely to have the acral or mucosal subtypes that often lack BRAF mutation.^9^, ^40^, ^41^

The COMBI‐v trial compared Dab/Tram with the BRAF inhibitor vemurafenib,^42^ and the COMBI‐d trial compared Dab/Tram with the BRAF inhibitor dabrafenib.^43^, ^44^, ^45^ They found that Dab/Tram significantly prolonged survival and had fewer skin‐related toxicities, including the development of squamous cell carcinoma in patients with advanced melanoma harboring the BRAF V600E/K mutation. The development of squamous cell carcinoma was seen in 18% of the patients in the vemurafenib‐treatment arm, whereas it was seen in only 1% of patients in the Dab/Tram‐treatment arm in the COMBI‐v trial. For first‐line Dab/Tram therapy, the 5‐year PFS and OS for the combined Dab/Tram treatment arms of the COMBI‐v and COMBI‐d trials were 19% and 34%, respectively^46^ (Table 1). As one in five patients who received Dab/Tram did not exhibit disease progression over 5 years, long‐term survival is expected for patients taking BRAF/MEKi, especially under favorable conditions such as good performance status, limited number of metastatic organs, low total volume of metastatic lesions, and normal LDH.^47^, ^48^

The COLUMBUS study evaluating Enco/Bini had two stages: encorafenib (450 mg) plus binimetinib (COMBO450) versus encorafenib alone or vemurafenib alone in Part 1, and encorafenib (300 mg) plus binimetinib (COMBO300) versus encorafenib alone in Part 2.^49^, ^50^, ^51^, ^52^, ^53^, ^54^ The 7‐year PFS and OS for first‐line Enco/Bini (COMBO450) treatment in Part 1 were 21% and 27%, respectively^54^ (Table 1). As more than one in five patients who received Enco/Bini did not experience disease progression for 7 years, long‐term survival can be expected with Enco/Bini in selected patients. However, the 5‐year and 7‐year OS rates of Enco/Bini in the COLUMBUS trial were 34.7% and 27.4% respectively, whereas the 5‐year and 7‐year OS rates of pembrolizumab in the KEYNOTE‐006 trial were 39.9% and 37.8%, respectively. Given that the difference between the 5‐year and 7‐year OS rates was 7.3% for Enco/Bini and only 2.1% for pembrolizumab, immunotherapy seems to result in better long‐term survival.

## 3 OPTIMAL DURATION OF SYSTEMIC THERAPY

Before the development of ICIs and BRAF/MEKi, when the prognosis of advanced melanoma was dismal, there were few discussions on whether treatment should be continued after a long‐term response. However, more than 10 years have passed since the first ICI was approved and we now know that even patients with metastatic melanoma can achieve sufficient long‐term survival. Therefore, identifying the ideal duration of systemic therapy is currently a key clinical issue that needs investigating to avoid unnecessary health and financial burdens.^55^, ^56^, ^57^

### 3.1 Targeted therapy

Several retrospective studies have reported the clinical outcomes of targeted therapies after early discontinuation of BRAF/MEK inhibitors for advanced BRAF V600‐mutant melanoma.^58^, ^59^, ^60^ In a retrospective study using the multicenter skin cancer registry ADOReg of the Dermatologic Cooperative Oncology Group (DeCOG), 461 patients received first‐line BRAF or BRAF/MEK inhibitors, and 37 patients subsequently achieved CR. Of these, all 11 patients who continued therapy until the data cut‐off maintained a CR, whereas 22 of the 26 patients (85%) who discontinued therapy eventually relapsed. Patients who received BRAF‐targeted therapy for >16 months had significantly longer PFS than those who received BRAF‐targeted therapy for a shorter period.^58^ In another multicenter, retrospective study, 36 (38%) of 94 patients who discontinued targeted therapy during the progression‐free stage (67 with CR, 21 with PR, and two with stable disease) finally progressed after a median follow‐up of 42.9 months. The initial treatment duration was shorter in patients who exhibited disease progression (median 18.3 months) compared to that in patients who did not (median 34.6 months).^60^ Both studies reported a strong association between treatment duration and reduced risk of progression following treatment cessation. Although sustained disease control beyond the cessation of targeted therapy is possible in some cases, novel predictive biomarkers are necessary to identify patients who can safely discontinue targeted therapy. A case series of 13 patients with metastatic melanoma who stopped BRAF‐targeted therapy after CR showed that circulating tumor DNA (ctDNA) became detectable at disease recurrence, except for in brain‐only progression.^61^ Therefore, liquid biopsies that detect ctDNA as a predictive biomarker should be further investigated.

### 3.2 Immune checkpoint inhibitors

A durable response is more common with ICI compared with BRAF/MEKi. In a pooled analysis of phase II and III trials of Nivo/Ipi therapy, median PFS was similar between patients who discontinued treatment because of AEs (8.4 months) and those who did not (10.8 months) (hazard ratio [HR], 0.99; 95% confidence interval [CI], 0.72–1.37).^62^, ^63^ In the KEYNOTE‐001 trial, an open‐label, phase 1b trial that included multiple cohorts of patients with advanced solid tumors including melanoma and non‐small cell lung cancer (NSCLC), sustained CRs were observed after discontinuation of pembrolizumab. Of the 105 patients who achieved CR, 67 discontinued pembrolizumab without additional anticancer therapy, and the 2‐year disease‐free survival rate after CR was 89.9%.^64^

In the CheckMate 153 trial for advanced NSCLC, patients who continued to receive nivolumab treatment for 1 year were randomly assigned to continue nivolumab (continuous group, *n* = 127) versus to stop nivolumab (1‐year fixed duration group, *n* = 125). After excluding patients who had progressed at the time of randomization, the median PFS was longer in the continuous group (*n* = 89) versus the 1‐year fixed duration group (*n* = 85) (24.7 months vs. 9.4 months; HR, 0.56; 95% CI 0.37–0.84).^65^ However, the primary endpoint of the CheckMate 153 trial was safety, and efficacy was an exploratory endpoint. In contrast, several retrospective studies on melanoma have reported clinical outcomes after early discontinuation of ICI therapy.^66^, ^67^, ^68^ In a real‐world analysis of 185 patients with advanced melanoma who discontinued anti‐PD‐1 treatment in the absence of disease progression or treatment‐limiting toxicity, 145 (78%) patients remained free from disease progression. Relapse after treatment discontinuation was infrequent in patients who achieved a CR and were treated for more than 6 months.^66^ Similarly, a multicenter, retrospective study in Japan with 57 patients who achieved a CR to anti‐PD‐1 monotherapy showed similar PFS after CR between the continuation group (*n* = 21) and the discontinuation after >6 months of therapy group (*n* = 25); however, poor PFS was observed in the discontinuation after ≤6 months of therapy group (*n* = 11). Importantly, this study included 12 patients with the acral subtype and 22 with the mucosal subtype, therefore, a sustained clinical benefit may occur regardless of the melanoma subtype.^68^

Currently, there are several ongoing clinical trials to determine the optimal duration of ICI treatment for advanced melanoma (Table 2). The STOP‐GAP trial in Canada is a randomized phase III trial comparing intermittent anti‐PD‐1 therapy with standard continuous anti‐PD‐1 therapy for up to 2 years. Patients randomized to the experimental arm will discontinue treatment after maximal tumor response, which is determined by at least two radiological assessments 3 months apart (NCT02821013).^69^ The Safe Stop trial in the Netherlands is a single‐arm, prospective study assessing whether early discontinuation of first‐line anti‐PD‐1 therapy is safe in patients with advanced melanoma who achieve a radiological response. Patients will discontinue treatment if radiological CR or progesterone partial response (PR) is confirmed (NL7293).^70^ The DANTE trial in the UK is a randomized phase III, non‐inferiority trial. Patients with advanced melanoma who have received first‐line anti‐PD‐1 therapy alone or in combination with anti‐CTLA‐4 for 12 months and are progression‐free will be randomized to continue anti‐PD‐1 therapy or to discontinue treatment (ISRCTN15837212).^71^ The PET‐Stop trial in the US is a single‐arm, phase II, biomarker‐driven trial that aims to assess how well 18 F‐fluorodeoxyglucose positron emission tomography‐computed tomography (FDG‐PET/CT) imaging can guide the early discontinuation of anti‐PD‐1 therapy in patients with advanced melanoma. After 1 year of anti‐PD‐1 therapy alone, or in combination with anti‐CTLA‐4, patients will receive an FDG‐PET/CT scan. Patients with a negative FDG‐PET/CT or positive FDG‐PET/CT scan but with a negative biopsy for viable tumors, will discontinue anti‐PD‐1 therapy and undergo active surveillance (Arm A). Patients with a positive FDG‐PET/CT scan and positive biopsy for a viable tumor or a positive FDG‐PET/CT scan and who did not receive a biopsy will continue anti‐PD‐1 for 12 months in the absence of disease progression or unacceptable toxicity (Arm B) (NCT04462406).^72^

**TABLE 2:** Ongoing prospective trials to determine the optimal duration of immune‐checkpoint inhibitor therapy for advanced melanoma.

Clinical trial	Country	Design	*N*	Interventions	Primary endpoint	Trial identifier
STOP‐GAP	Canada	Randomized	614	Stop at MTR	OS	NCT02821013
Safe Stop	The Netherlands	Single arm	200	Stop at CR or PR	Ongoing response at 2 years	NL7293
DANTE	UK	Randomized	1208	Stop if progression‐free at 1 year	PFS	ISRCTN15837212
PET‐Stop	USA	Single arm	150	Stop if PET negative or PET positive but with negative biopsy	EFS	NCT04462406

Abbreviations: CR, complete response; EFS, event‐free survival; MTR, maximal tumor response; OS, overall survival; PET, positron emission tomography; PFS, progression free survival; PR partial response.

## 4 EMERGING COMBINATION THERAPIES

The current standard of care for systemic therapy of advanced melanoma is anti‐PD‐1‐based immunotherapy. Currently, Nivo/Ipi has the best long‐term results; however, a high dose of ipilimumab (3 mg/kg) can result in a high frequency of irAEs. Therefore, the development of combination therapy with a better risk–benefit balance is highly needed. Trials with this aim include the KEYNOTE‐252 trial, which assessed a combination of epacadostat, an IDO1 inhibitor, and pembrolizumab;^73^ the COMBI‐i trial, which assessed the combination of Dab/Tram and spartalizumab, an anti‐PD‐1 antibody;^74^ the MASTERKEY‐265 trial, which assessed a combination of T‐VEC, an oncolytic virus, and pembrolizumab;^75^ and the PIVOT IO 001 trial, which assessed a combination of bempegaldesleukin, pegylated‐IL‐2, and nivolumab.^76^ However, none of these combinations were superior to anti‐PD‐1 monotherapy or BRAF/MEKi (Table 3). In the IMspire150 trial, a combination of vemurafenib plus cobimetinib (Vem/Cobi), a BRAF/MEKi, and atezolizumab, an anti‐PD‐L1 antibody, was compared with Vem/Cobi in 514 treatment‐naïve patients with advanced BRAF V600‐mutant melanoma. Although the investigator‐assessed PFS was prolonged in the triplet combination treatment arm, it was not confirmed by the central review, and OS was similar between the treatment arms.^77^, ^78^, ^79^

**TABLE 3:** Randomized phase III trial of anti‐PD‐1/PD‐L1‐based combination therapy for advanced melanoma.

Class	Experimental arm vs. control arm	*N*	Primary endpoint	Mucosal eligible?	PFS HR, (95% CI)	OS HR, (95% CI)	Result	Ref
Clinical trial								
*PD‐1/CTLA‐4*								
CheckMate 067	(A) Nivo (1)/Ipi (3) (B) Nivo (3) vs. Ipi (3)	314 316 315	PFS, OS	Yes	A: 0.42 (0.35–0.51) B: 0.53 (0.44–0.64) A vs. B^b^: 0.79 (0.65–0.97)	A: 0.53 (0.44–0.65) B: 0.63 (0.52–0.77) A vs. B^b^: 0.84 (0.68–1.04)	Pos	15, 16, 17, 18, 19
CheckMate 511	Nivo (3)/Ipi (1) vs. Nivo (1)/Ipi (3)	180 178	TRAEs	Yes	1.06 (0.79–1.42)^b^	1.09 (0.73–1.62)^b^	Pos^c^	22
*PD‐1/LAG‐3*								
Relativity‐047	Nivo/Rela vs. Nivo	355 359	PFS	Yes	0.81 (0.67–0.97)	0.82 (0.67–1.02)	Pos	80, 81, 82
PD‐1/IDO1								
KEYNOTE‐252^d^	Pembro/Epacado vs. Pembro	354 352	PFS, OS	Yes	1.00 (0.83–1.21)	1.13 (0.86–1.49)	Neg	73
*PD‐1/IL‐2*								
PIVOT IO 001	Nivo/BEMPEG vs. Nivo	391 392	ORR, PFS, OS	Yes	1.09 (0.88–1.35)	0.94 (0.59–1.48)	Neg	76
*PD‐1/Oncolytic virus*								
MASTERKEY‐265	Pembro/T‐VEC vs. Pembro	346 346	PFS, OS	No	0.86 (0.71–1.04)	0.96 (0.76–1.22)	Neg	75
*PD‐1/BRAF/MEKi*								
COMBI‐i^d^	Sparta/Dab/Tram vs. Dab/Tram	267 265	PFS	No	0.82 (0.66–1.03)	0.79 (0.59–1.05)	Neg	74
*PD‐L1/BRAF/MEKi*								
IMspire150	Atezo^a^/Vem/Cobi vs. Vem/Cobi	255 246	PFS	Yes	0.79 (0.64–0.97)	0.84 (0.66–1.06)	Pos	77, 78, 79

Abbreviations: Atezo, atezolizumab; BEMPEG, bempegaldesleukin; BRAF/MEKi, BRAF/MEK inhibitors; CI, confidence interval; CTLA‐4, anti‐CTLA‐4 antibody; Dab/Tram, dabrafenib/trametinib; Epacado, epacadostat; HR, hazard ratio; IDO1, indoleamine 2,3‐dioxygenase 1; Ipi (1), ipilimumab 1 mg/kg; Ipi (3), ipilimumab 3 mg/kg; LAG‐3, anti‐LAG‐3 antibody; N, number of the patients; Neg, negative; Nivo (1), nivolumab 1 mg/kg; Nivo (3), nivolumab 3 mg/kg; ORR, objective response rate; OS, overall survival; PD‐1, anti‐PD‐1 antibody; Pembro, pembrolizumab; PFS, progression‐free survival; Pos, positive; Ref, reference; Rela, relatlimab; Sparta, spartalizumab; TRAEs, the rate of treatment‐related grade 3 to 5 adverse events; T‐VEC, talimogene laherparepvec; Vem/Cobi, vemurafenib/cobimetinib.

^a^ Vem/Cobi alone in the 28‐day run‐in period.

^b^ Descriptive analysis.

^c^ Positive for safety.

^d^ Participants from Japan.

In the RELATIVITY‐047 trial, a fixed‐dose combination of nivolumab plus relatlimab, (relatlimab‐nivolumab), an anti‐LAG‐3 antibody, was compared with nivolumab monotherapy. A total of 714 treatment‐naïve patients with advanced melanoma were included in this study. The primary endpoint was PFS, and a significantly longer PFS with relatlimab‐nivolumab was demonstrated (median PFS, 10.1 vs. 4.6 months, and HR 0.75 [95% CI 0.62–0.92]). The frequency of grade 3 or greater treatment‐related AEs of relatlimab‐nivolumab and nivolumab monotherapy were 18.9% and 9.7%, respectively.^80^, ^81^, ^82^ Relatlimab‐nivolumab was approved by the Food and Drug Administration and the European Medicines Agency in 2022 but has not been approved in Japan because Japan did not participate in the RELATIVITY‐047 trial. Japan participated only in the RELATIVITY‐020 trial evaluating second‐line relatlimab‐nivolumab.^83^ Relatlimab‐nivolumab is expected to replace anti‐PD‐1 monotherapy as the standard of care; however, it is unclear whether relatlimab‐nivolumab will replace Nivo/Ipi therapy. In terms of irAEs, relatlimab‐nivolumab was superior to Nivo/Ipi, with a frequency of 18.9% grade 3 or higher AEs compared with 59% for Nivo/Ipi therapy. In terms of long‐term survival, the median OS of patients who received Nivo/Ipi was 72.1 months, indicating that long‐term survival is expected, whereas the follow‐up period for relatlimab‐nivolumab is still short, and long‐term results have not yet been reported.

Recently, the NIPPON trial (JCOG2007, jRCTs031210013), a randomized phase III trial evaluating Nivo/Ipi plus platinum‐based chemotherapy regimen for treatment‐naive patients with advanced NSCLC, was terminated early because of a high proportion of treatment‐related deaths.^84^, ^85^ The dose of ipilimumab used in the NIPPON trial was 1 mg/kg every 6 weeks, which was lower than that for advanced melanoma. Given this, developing a new combination therapy by adding another agent to Nivo/Ipi seems difficult. Currently, there are several clinical trials of anti‐PD‐1/L1‐based combination therapy for advanced melanoma including histone deacetylase inhibitor, tumor‐infiltrating lymphocytes therapy, vaccine therapy, vascular endothelial growth factor‐targeting therapies, preferentially expressed antigen in melanoma (PRAME)‐targeted bispecific proteins, T‐cell receptor‐T targeting PRAME, and more. Ongoing randomized phase III and Japanese phase II trials are listed in Table 4. Although the primary endpoints of these trials include PFS and objective response rate, long‐term clinical outcomes should also be evaluated to assess the benefits of novel agents.

**TABLE 4:** Ongoing randomized phase III and Japanese phase II trials of anti‐PD‐1/L1‐based combination therapy for advanced melanoma.

Class	Phase	Experimental arm	Control arm	Line	*N*	Mucosal eligible?	Primary endpoint	Estimated primary completion	Trial identifier
GM‐CSF	II/III	Nivo/Ipi/GM‐CSF	Nivo/ Ipi	1st	600	No	OS	2033	NCT02339571
TLR9	II/III	Nivo/CMP‐001	Nivo	1st	450	No	ORR, PFS	2024	NCT04695977
HDACi^b^	III	Nivo/HBI‐8000	Nivo	1st	480	Yes	ORR, PFS	2024	NCT04674683
TACE	III	Pucotenlimab/TACE	TMZ/ TACE, Pembro	1st	350	Yes	PFS, OS	2024	NCT05647954
LAG‐3	III	Cemiplimab/ Fianlimab	Pembro	1st	1590	Yes	PFS	2025	NCT05352672
Vaccines	III	Pembro/IO102‐IO103	Pembro	1st	380	Yes	PFS	2025	NCT05155254
BRAF/MEKi	III	Pembro/Enco/Bini	Pembro	1st	624	No	ORR	2025	NCT04657991
TIL	III	Pembro/ Lifileucel	Pembro	1st	670	Yes	ORR, PFS	2028	NCT05727904
PAI‐1i^a^	II	Nivo/TM5614	None	Any	40	Yes	ORR	Active, not recruiting	jRCT2021210029
KITi^a^	I/II	Pembro/ Imatinib	None	2nd	22	Yes	ORR	Recruiting	jRCTs031190202
PARPi^a^	II	Nivo, Pembro/ Niraparib	None	2nd	57	Yes	ORR	Recruiting	jRCT2051210120
Oncolytic virus^a^	I/II	Nivo/T‐hIL12	None	1st	24	Yes	ORR	Recruiting	jRCT2033190086

Abbreviations: BRAF/MEKi, BRAF/MEK inhibitors; Enco/Bini, encorafenib/binimetinib; GM‐CSF, granulocyte‐macrophage colony‐stimulating factor; HDACi, histone deacetylase inhibitor; KITi, KIT inhibitor; LAG‐3, anti‐lymphocyte‐activation gene 3 antibody; N, number of the patients; Nivo, nivolumab; Nivo/Ipi, nivolumab/ipilimumab; ORR, objective response rate; OS, overall survival; PAI‐1i, inhibitor of plasminogen activator inhibitor‐1; PARPi, PARP inhibitor; Pembro, pembrolizumab; PFS, progression‐free survival; TACE, transcatheter arterial chemoembolization; TIL, tumor‐Infiltrating lymphocyte therapy; TLR9, toll‐like receptor 9 agonist; TMZ, temozolomide.

^a^ Japanese clinical trial.

^b^ Participated from Japan.

## 5 CONCLUSION

Currently, long‐term survival of melanoma is most likely to be achieved with the use of the standard‐dose regimen of Nivo/Ipi combination therapy. However, Nivo/Ipi is associated with a high frequency of serious or persistent irAEs. Several new anti‐PD‐1‐based combination therapies with a better risk–benefit balance are under development. Although the acral and mucosal subtypes tend to be less responsive to ICIs, anti‐PD‐1‐based combination therapy should continue to be investigated for these subtypes owing to its potential for better long‐term survival. With the development of efficacious immunotherapy and targeted therapy, it is important to determine the optimal duration of systemic therapy to avoid unnecessary health and financial burdens as well as to improve efforts to support long‐term cancer survivors. As the goal of systemic therapy shifts from tumor control to long‐term survival, long‐term clinical outcomes should be evaluated to assess the benefits of novel agents in future clinical trials.

## FUNDING INFORMATION

This study did not receive any specific grants or technical support from funding agencies.

## CONFLICT OF INTEREST STATEMENT

Outside the submitted work, Kenjiro Namikawa received honoraria from Ono pharmaceutical, Novartis, Bristol‐Myers Squibb, and MSD, and served as an advisory board member for Novartis and MSD. Naoya Yamazaki received an institutional research grant from Ono Pharmaceutical, Novartis, Bristol‐Myers Squibb, and MSD, and served as an advisory board member for Ono Pharmaceutical, Chugai Pharma, MSD, Astellas Amgen BioPharma, Merck Serono, and Takara Bio, and received honoraria from Ono Pharmaceutical, Novartis, Bristol‐Myers Squibb, and MSD. All other authors have no conflicts of interest to declare.
